# ECG Scoring for the Evaluation of Therapy-Naïve Cancer Patients to Predict Cardiotoxicity

**DOI:** 10.3390/cancers13061197

**Published:** 2021-03-10

**Authors:** Julia Pohl, Raluca-Ileana Mincu, Simone M. Mrotzek, Reza Wakili, Amir A. Mahabadi, Sophia K. Potthoff, Jens T. Siveke, Ulrich Keller, Ulf Landmesser, Tienush Rassaf, Markus S. Anker, Matthias Totzeck

**Affiliations:** 1 West German Heart and Vascular Center, Department of Cardiology and Vascular Medicine, University Hospital Essen, Medical Faculty, 45147 Essen, Germany; J.Pohl@uk-essen.de (J.P.); Raluca-Ileana.Mincu@uk-essen.de (R.-I.M.); Simone.Mrotzek@uk-essen.de (S.M.M.); Reza.Wakili@uk-essen.de (R.W.); Amir-Abbas.Mahabadi@uk-essen.de (A.A.M.); Tienush.Rassaf@uk-essen.de (T.R.); 2 Department of Cardiology, Charité University Medicine Berlin (CBF), 12203 Berlin, Germany; sophia-katharina.potthoff@charite.de (S.K.P.); ulf.landmesser@charite.de (U.L.); markus.anker@charite.de (M.S.A.); 3 Berlin Institute of Health Center for Regenerative Therapies (BCRT), 13353 Berlin, Germany; 4 DZHK (German Center for Cardiovascular Research), Partner Site Berlin, 12203 Berlin, Germany; 5 West German Cancer Center, Institute for Developmental Cancer Therapeutics, University Medicine Essen, 45147 Essen, Germany; Jens.Siveke@uk-essen.de; 6 West German Cancer Center, Department of Medical Oncology, University Medicine Essen, 45147 Essen, Germany; 7 Division of Solid Tumor Translational Oncology, German Cancer Consortium (DKTK, Partner Site Essen) and German Cancer Research Center (DKFZ), 69120 Heidelberg, Germany; 8 Department of Hematology, Oncology, and Tumor Immunology, Campus Benjamin Franklin, Charité University Medicine Berlin, 12203 Berlin, Germany; Ulrich.Keller@charite.de; 9 German Cancer Consortium (DKTK), German Cancer Research Center (DKFZ), 69120 Heidelberg, Germany; 10 Max-Delbrück-Center for Molecular Medicine, 13125 Berlin, Germany; 11 Berlin Institute of Health (BIH), 10117 Berlin, Germany

**Keywords:** ECG, cardio-oncology, cancer, score, cardiotoxicity

## Abstract

**Simple Summary:**

Due to improved survival upon effective anti-cancer therapies, the management of treatment-related side-effects is of increasing interest and importance. Cardiovascular side-effects of chemo-, targeted- and/or immunotherapies are common and can be harmful. To date, the identification of patients who could experience those cardiovascular side-effects prior to the anti-cancer therapy start is difficult. We show that the use of a simple electrocardiographic (ECG) score can help to predict the occurrence of cardiovascular toxicity of anti-cancer therapies.

**Abstract:**

Objective: To evaluate a new electrocardiographic (ECG) score reflecting domains of electrical and structural alterations in therapy-naïve cancer patients to assess their risk of cardiotoxicity. Methods: We performed a retrospective analysis of 134 therapy-naïve consecutive cancer patients in our two university hospitals concerning four ECG score parameters: Contiguous Q-waves, markers of left ventricular (LV) hypertrophy, QRS duration and JTc prolongation. Cardiotoxicity was assessed after a short-term follow-up (up to 12 months). Results: Of all the patients (*n* = 25), 19% reached 0 points, 50% (*n* = 67) reached 1 point, 25% (*n* = 33) reached 2 points, 5% (*n* = 7) reached 3 points and 0.7% reached 4 or 5 points (*n* = 1 respectively). The incidence of cardiotoxicity (*n* = 28 [21%]) increased with the ECG score, with 0 points at 0%, 1 point 7.5%, 2 points 55%, 3 points 71% and ≥3 points 50%. In the ROC (Receiver operating curves) analysis, the best cut-off for predicting cardiotoxicity was an ECG score of ≥2 points (sensitivity 82%, specificity 82%, AUC 0.84, 95% CI 0.77–0.92, *p* < 0.0001) which was then defined as a high-risk score. High-risk patients did not differ concerning their age, LV ejection fraction, classical cardiovascular risk factors or cardiac biomarkers compared to those with a low-risk ECG score. Conclusion: ECG scoring prior to the start of anti-cancer therapies may help to identify therapy-naïve cancer patients at a higher risk for the development of cardiotoxicity.

## 1. Introduction

Risk prediction for cardiotoxicity in cancer patients is an important aspect in the field of cardio-oncology. Several parameters have previously been associated with an increased risk for cardiovascular adverse events from cancer therapy including age, sex, pre-existing cardiac diseases (especially left ventricular (LV) dysfunction and coronary artery disease (CAD)), arterial hypertension, specific chemotherapy drugs and regimens, their cumulative dose and previous or concomitant chemo- and/or radiation therapies [1,2,3,4,5]. Baseline evaluation of cardio-oncology patients includes patient history, physical examination, cardiac biomarkers including high-sensitive Troponin I or T (hsTnI or hsTnT) and the N-terminal fragment of the pro brain natriuretic peptide (NT-proBNP), echocardiography to assess LV function and global longitudinal strain (GLS) and electrocardiographic (ECG) analysis. ECG analysis is a recommended component of each cardio-oncological evaluation, because malignant arrhythmias are frequent side effects of many cancer therapies (e.g., anthracyclines, small-molecule tyrosine kinase inhibitors, Bruton’s tyrosine kinase inhibitors, immune checkpoint inhibitors and B-Raf proto-oncogene serine/threonine-kinase (BRAF) inhibitors) [3,6,7,8]. Early identification of ECG abnormalities associated with structural or electrical alterations is therefore of great importance. This may relate to pathologic contiguous Q waves, ECG markers of LV hypertrophy and left atrial enlargement, increased heart rate and heart rate variability, prolongation of the PR and QRS intervals, QRS fragmentations, left bundle branch blocks, JTc prolongation and contiguous T-wave inversion, which were shown to be associated with cardiac adverse events in the general population [9,10,11,12,13,14]. The role of ECG parameters in the prediction of mortality in cancer patients has been assessed previously [15,16]. Interestingly, childhood-cancer survivors with previous anthracycline-treatments showed a broad range of ECG abnormalities including pathological Q waves and signs of LV hypertrophy, even 20 years after the initial diagnosis and treatment. These ECG abnormalities were predictive of cardiac and all-cause mortality [17]. Medical scores can help to support decision making and patient management. They can also help to predict the probability of several conditions, to assess risks and the severity of conditions and to diagnose diseases accurately [18]. In cardio-oncology, scoring could help to identify at-risk patients independently or with respect to their planned chemotherapy regimen and to individualize follow-ups and cardioprotective therapies. Risk assessment by scoring systems is a part of personalized precision medicine and therefore should be part of modern cardio-oncology. More recently, a simple ECG score was developed to improve the prediction of sudden and/or arrhythmic death (SAD) in patients with CAD [19]. The authors analyzed the data of more than 7000 patients from a large registry database (PRE-DETERMINE) and a prospective study (ARTEMIS) to develop a simple ECG score composed of four ECG measures incorporated into a standard 12-lead ECG analysis. The score distinguished between three risk groups (low-, moderate- and high-risk groups) and was shown to be associated with the risk of SAD as well as the risk of non-SAD, although this association was weaker. Whether the ECG score developed for CAD patients can also be used to assess a group of therapy-naïve cancer patients is unclear. We aim to characterize this newly developed ECG score in a therapy-naïve cancer patient collective and show follow-up data concerning the occurrence of cardiotoxicity after anti-cancer therapy initiation.

## 2. Materials and Methods

This retrospective investigation included 134 consecutive therapy-naïve cancer patients who were examined in our two cardio-oncology units (the West German Heart and Vascular Center, Essen and the Department of Cardiology, Charité Campus at Benjamin Franklin, Berlin) between 2018 and 2019 before the start of chemo- and/or immunotherapy (in- and exclusion criteria: Table S1). All patients underwent the same standard clinical examinations with anamnesis, physical examination, ECG, echocardiography and laboratory analysis including the cardiologic biomarkers hsTnI and NT-proBNP. Anthropometric measurements (height, weight) were assessed in all patients, and body mass index (BMI) and body surface area (BSA) were calculated accordingly. Data regarding different factors such as hypertension, atrial fibrillation and premedication were acquired retrospectively from medical records. Follow-up data concerning the occurrence of cardiotoxicity after chemo- and/or immunotherapy start during the follow-up period of 3–12 months (depending on the planned therapy regimen) were taken from medical records. The study was approved by the local ethics committees.

Cardiotoxicity was diagnosed if the LV function decreased by ≥10% to <50% or if the global longitudinal strain (GLS) decreased by 15% according to current recommendations [1,6].

**Figure 1:**
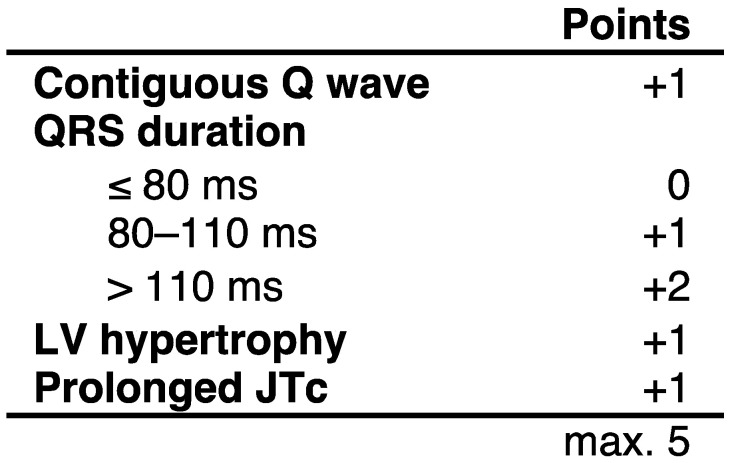
Composition of the ECG score. The table shows the composition of the ECG score developed by Chatterjee et al. [19].

Twelve-lead electrocardiograms (ECGs) were recorded using GE Healthcare ECG machines (GE, Milwaukee, WI, USA). Assessment of the ECG score was done in accordance with the original publication in which the score was developed (Figure 1) [15]. In brief, all ECGs were evaluated concerning the following parameters: contiguous Q wave, QRS duration, marker of LV hypertrophy and prolongation of the JTc interval. Contiguous Q waves were defined as a Q wave duration > 40 ms and/or a Q:R ratio amplitude > 25% in any two contiguous leads [16]. The presence of a contiguous Q wave counted as 1 point. The QRS duration was measured in a standard manner in lead II. A QRS duration of <80 ms was yielded for 0 points, 80–110 ms for 1 point and >110 ms for 2 points. The LV hypertrophy was assessed by the Sokolow−Lyon Index being positive (+1 point) if the sum of S in V1 and R in V5 or V6 was ≥3.5 mV [17]. The JTc time was calculated by QTc minus the QRS duration with QT being measured as the maximum value in leads II, V5 or V6 and QTc being calculated with Bazzett’s formula [11]. A JTc prolongation > 360 ms accounted for 1 point. After assessment of all 4 parameters, the ECG score was calculated by summation (Figure 1) [19].

Continuous variables are shown as the mean ± standard deviation (SD). Normal distribution was tested by the D’Agostino & Pearson omnibus normality test. Two independent groups were compared using the student’s t-test for normally distributed variables and using the Mann–Whitney U test for nonparametric parameters. Receiver operating curves (ROC) were determined to analyze the predictive potential of the ECG score concerning cardiotoxicity, and the area under the curve (AUC) was calculated. The Youden index was calculated to identify the ECG score cut-off value for cardiotoxicity and proportions were compared using the Chi-square test. Correlations between parameters were analyzed by Spearman rank correlation and multiple testing was corrected by the Bonferroni correction. Sensitivity analysis was performed using Fisher’s exact test. Data were analyzed using Prism software (version 8.4, GraphPad software, San Diego, CA, USA). A *p*-value < 0.05 was considered statistically significant.

## 3. Results

### 3.1. Baseline Characteristics

The baseline characteristics of the study population are provided in Table 1. The mean age of the patients was 62 ± 14 years, and 68% of the patients were female. All patients were naïve with regard to systemic therapy and presented in our cardio-oncology departments for routine examination before the start of anti-cancer therapy. The patients suffered from skin cancer (48%), breast cancer (23%), leukemia (5%) and lymphoma (15%). Most of the patients were in advanced stages III (27%) and IV (39%) according to the UICC (Union internationale contre le cancer)/TNM or the Ann-Arbor (lymphoma) staging system. The majority of the patients had a normal LV ejection fraction (LV-EF) (>50%; mean 59 ± 7%). Most of the patients also had normal hsTnI (interquartile range (IQR) 0–7 ng/L, Table 1) and NT-proBNP values (IQR 63–325 pg/mL, Table 1). Arterial hypertension was the most common cardiovascular risk factor, followed by smoking and dyslipidemia. The corresponding intake of anti-hypertensive drugs is listed in Table 1. Only 15% of the patients had a history of CAD (*n* = 18).

### 3.2. Estimation of ECG Score

**Figure 2:**
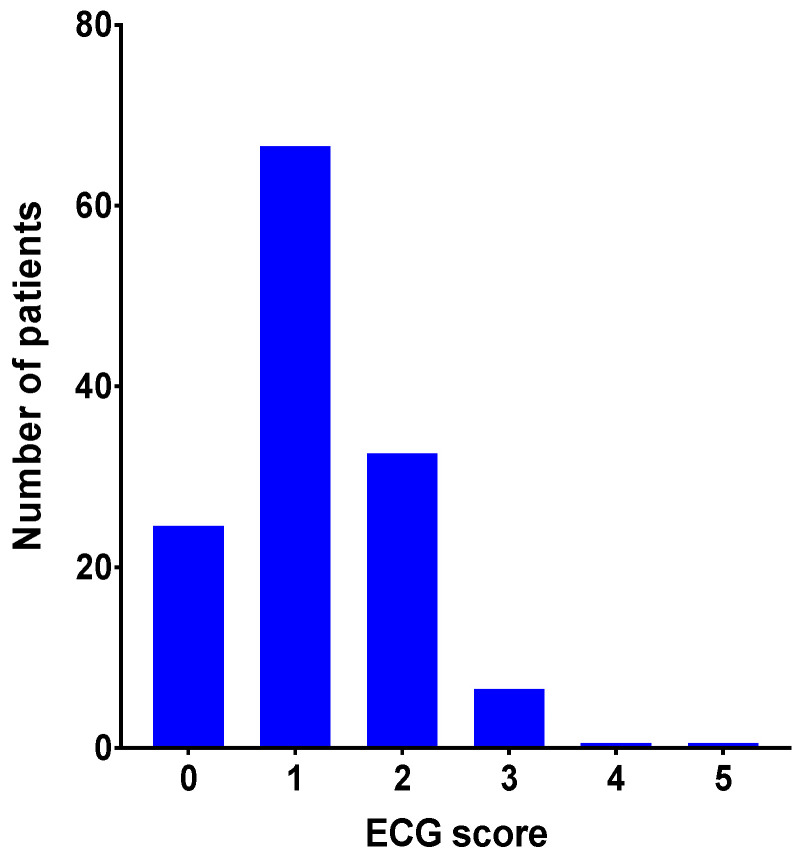
Distribution of risk groups and risk-based electrocardiographic (ECG) score: 19% of the patients (total *n* = 25) had an ECG score of 0 points, 50% (*n* = 67) 1 point, 25% (*n* = 33) 2 points, 5% (*n* = 7) 3 points and 0.7% (*n* = 1) 4 or 5 points respectively.

The ECG score was calculated in all 134 patients [19] and 25 patients achieved 0 points, 67 patients achieved 1 point, 33 patients achieved 2 points, seven patients achieved 4 points and one achieved 4 or 5 points respectively (Figure 2).

Contiguous Q waves were present in 4% of the patients, 18% showed a QRS duration of ≤80 ms, 69% 80–110 ms and 13% had a QRS duration > 110 ms. ECG signs of LV hypertrophy showed in 13% of the patients and a JTc duration of >360 ms was seen in 28% of the study patients (Table 2).

### 3.3. The ECG Score Predicts Cardiotoxicity

**Figure 3:**
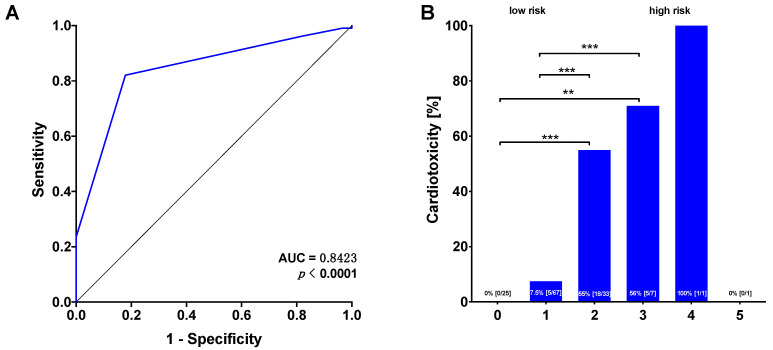
(**A**) Predictive value of the ECG score for cardiotoxicity determined using the ROC analysis. (**B**) Incidences of cardiotoxicity increase with increasing score values. ** *p* < 0.01, *** *p* < 0.001.

The incidence of cardiotoxicity in the whole study cohort was 21%. The incidence of cardiotoxicity during the follow-up was 0% in patients with 0 points in the ECG score (0/25), 7.5% in patients with 1 point (5/67), 55% in patients with 2 points (18/33), 71% in patients with 3 points (5/7), 100% in the 4-points patient (1/1) and 0% in the patient with 5 points (0/1) (Figure 3A). The sensitivity was 0.82 (95% CI 0.74–0.88) and the specificity was 0.82 (95% CI 0.64–0.92) with a positive predictive value of 0.95 (95% CI 0.88–0.98) and a negative predictive value of 0.55 (95% CI 0.40–0.69) (Table 3). The ROC analysis revealed an area under the curve of 0.8432 (95% CI 0.77–0.92; *p* < 0.0001, Figure 3B) for the prediction of cardiotoxicity. The Youden index identified ≥ 2 points as the best cut-off value for cardiotoxicity. Therefore, we defined two groups concerning their risk of developing cardiotoxic side-effects: patients with an ECG score < 2 were assigned to the low-risk group and patients with an ECG score ≥ 2 were assigned to the high-risk group. The incidence of cardiotoxicity was 5.4% in the low-risk group and 57.1% in the high-risk group (*n* = 92/42, *p* < 0.0001).

The two risk groups did not differ concerning age, sex, LV-EF, or laboratory values, e.g., hemoglobin, platelet count, creatinine, hsTnI and NT-proBNP (Table 4). C-reactive protein (CRP) was higher in the low-risk group (7.6 ± 2.0 mg/dL vs. 2.2 ± 4.7 mg/dL, *n* = 92/42, *p* < 0.05, Table 4) and the BMI was higher in the high-risk patients (29.1 ± 5.8 vs. 26.0 ± 4.4 in low-risk group, *n* = 42/92. *p* < 0.01, Table 4). High-risk patients were treated with beta-blockers more frequently compared to the other group (57% vs. 29%, *n* = 42/92, *p* < 0.01, Table 4). There was a strong trend for a higher prevalence of treatment with ASA without being statistically significant. The distribution of patients with CAD was not statistically different amongst the groups (12% vs. 17%, *n* = 92/42, *p* = ns, Table 4).

### 3.4. The ECG Score Is Independent of Classical Factors of Cardiotoxicity

The Spearman rank correlation showed that the ECG score was not associated with age, sex, BMI, heart rate, presence of classical cardiovascular risk factors, intake of betablockers or ACE-I/ARB, hsTnI and NT-proBNP or LV-EF (Table 5). There was a weak association with the NYHA class (r = 0.2579, *p* = 0.0435, Table 5).

## 4. Discussion

We have presented the descriptive data on a simple ECG score by Chatterjee et al. in therapy naïve-cancer patients [19]. We showed that a higher score is associated with a higher incidence of cardiotoxicity and that an ECG score ≥ 2 has good discriminatory power to predict cardiotoxicity in the ROC analysis. The score was independent of age, the presence of CAD, arterial hypertension or atrial fibrillation, LV-EF or cardiac biomarkers.

The original score was developed to predict the incidence of SAD in CAD patients, but the single parameters of this ECG score represent structural and electrical alterations which are not specific for ischemic heart disease and could therefore be translated to several cardiac abnormalities or potentially cardiotoxic interventions. Some of these parameters were already validated in heart failure patients without ischemic heart disease [20], while others were predictive of SAD in the general population [14,21,22,23,24]. Of course, the collective examined here is not comparable to CAD patients. Although most of our patients were free of pre-existing CAD, the distribution of the score in our population was comparable to the score distribution in the CAD cohort, with most of the patients having 0–1 point (69% (our data) vs. 55/61% (study cohorts)), circa one-fourth to one-third achieving 2 points (25% (our data) vs. 31/29% (study cohorts)) and only a few patients having ≥3 points (7% (our data) vs. 14/10% (study cohorts)). Whether the risk for SAD or other events in these patients is comparable to the risk of CAD patients remains unclear and the present study did not aim at performing a mortality analysis.

This study is the first to evaluate an ECG-based score concerning therapy-naïve cancer patients and its potential to predict cardiotoxicity. High ECG scores were associated with high incidences of cardiotoxicity. Higher patient numbers would be necessary to validate these results for the group with four or five points. Although the score was designed and validated for the prediction of mortality in CAD patients, contingency analyses and receiver operating curves showed high sensitivity, specificity and accuracy to predict cardiotoxicity in the present study (Table 5). While the advantages of the ECG score are its easy calculation and cost-effectiveness, other clinical risk scores are more complex and include parameters like age, cardiovascular risk factors and chemo-/immunotherapy doses [25,26,27].

In this study, 21% of the patients had cardiotoxic side-effects of their anti-cancer therapies. This rate seems high but can be explained by two reasons. First, many patients were treated with anthracyclines, many of them in combination with alkylating agents and furthermore showed other factors of higher rates of cardiotoxicity such as female sex, preexisting arterial hypertension and higher ages (Tables S2 and Table 1). We included a large group of skin cancer patients treated with immune-checkpoint inhibitors. The cardiovascular side-effects of those therapies were described as being rare but seemed to be of higher incidence [28,29]. Second, they were consecutive patients referred to our cardio-oncology departments by their treating physicians due to preexisting cardiovascular risk-factors, diseases or symptoms. Thereby, higher rates of cardiotoxicity seem to be reasonable.

The ECG score was not associated with markers of cardiac damage or volume overload like hsTnI and NT-proBNP before the start of anti-tumor therapies. This could be due to the fact that most of the patients had normal biomarker levels at presentation. The mean hsTnI value was 6 ng/L with only 3% of the patients having an hsTnI level above the upper limit of normal (Table 1). The role of cardiac biomarkers in cardio-oncology has been discussed intensively and controversially. The assessment of troponins and NT-proBNP is recommended for patients receiving anthracyclines by the American Society of Clinical Oncology (ASCO) [30]. The European Society of Medical Oncology (ESMO) guidelines recommend the measurement of troponins before and during therapies with any kind of potentially cardiotoxic therapy, while the European Society of Cardiology (ESC) has not recommended a standardized approach in cancer patients as yet [1,30]. Elevation of troponins is associated with an overall increase of cardiovascular adverse events and mortality [31]. Troponins were shown to be useful for the detection of acute cardiotoxicity representing acute myocardial damage in settings of high-dose chemotherapies and immune checkpoint inhibitory therapies [2,32,33]. Elevated troponins predict LV dysfunction in chemotherapy patients, and therefore, can be used as a tool to diagnose cardiotoxicity [17].

The present study has several limitations. The first is the fact that 13–18% of the patients showed classical cardiovascular risk factors, e.g., diabetes or smoking, while half of the patients suffered from preexisting arterial hypertension and were being treated accordingly (Table 1). This may be interpreted as a “real world collective” in cardio-oncology departments, and comparable patient collectives in cardio-oncology have been described before [32]. This can also be related to the retrospective design including all consecutive patients presenting in our cardio-oncology departments. However, the Spearman rank correlation did not show any association between classical cardiovascular risk factors or premedication and the ECG score (Table 5). The next limitation related to the study population with a multitude of different cancer entities and therapies. Again, this reflects the daily practice in dedicated cardio-oncology departments, but we are aware that the tumor biology can play a role in the origin of cardiac complications as well as anti-tumor therapies. Nonetheless, we aimed at investigating a score which can be calculated easily by each physician using ECG parameters only. This score has not been applied to other study collectives than the CAD patients from the initial publication, but our study implies that it can be potentially useful to predict cardiotoxicity in cancer patients. It is tempting to speculate that the score can be used to also predict cardiovascular events in other collectives. Further studies must assess whether the addition of parameters, e.g., anthracycline doses, tumor type, age, LV-EF etc. to the ECG score could further improve the prognostic accuracy of the score, especially for collectives like breast cancer patients undergoing high-dose anthracycline or anti-Her2 therapies.

Although the study results are further limited by the retrospective design and although relatively low patient numbers of only *n* = 134 were included, the results show good values for sensitivity and AUC.

## 5. Conclusions

We have reported that around one-third of typical therapy-naïve cancer patients presenting in cardio-oncology are at a high risk of developing cardiotoxicity according to a newly developed ECG score. The evaluation and consideration of this new ECG score in the setting of cardio-oncology could be helpful to identify patients at a higher risk of developing cardiotoxic side effects. Although our data is hypothesis-generating, we think that the assessment of the ECG score may be a new component of personalized precision medicine in cardio-oncology. Accurate tools for the prediction of cardiotoxicity could, e.g., shorten the follow-up routines for high-risk patients or lead to an early start of a preventive drug therapy in those patients. Prospective long-term data are needed to confirm reliable factors for the prediction of cardiotoxicity in cancer patients.

## Figures and Tables

**Table 1:** Baseline Characteristics.

Parameters	Value
Age (years)	62 ± 15
Female sex (%w)	68
BMI (kg/m^2^)	27.0 ± 5.0
Heart rate (bpm)	74 ± 13
Hemoglobin (g/dL)	12.7 ± 1.9
Platelets (×1000/µL)	290 ± 106
Creatinine (mg/dL)	0.9 ± 0.3
CRP (mg/dL)	5.9 ± 6.5
hsTnI (ng/L), (IQR)	6 (0–7)
NT-proBNP (pg/mL), (IQR)	565 (63–325)
NYHA class (I/II/III/IV) (%)	65/29/6/0
Comorbidities	
Arterial hypertension (%)	54
Diabetes (%)	14
Dyslipidemia (%)	13
Smoking (%)	18
Previous stroke (%)	18
CKD	7
Atrial fibrillation (%)	8
Known CAD (%)	15
Premedication	
ACE-I/ARB (%)	44
Betablocker (%)	38
ASA (%)	31
DOAC (%)	9
Tumor stadium (UICC or Ann-Arbor classification for lymphoma)	
I (%)	7
II (%)	25
III (%)	27
IV (%)	39
Tumor entity	
Skin cancer (%)	48
Breast cancer (%)	23
Lymphoma (%)	15
Leukemia (%)	5
others (%)	9

BMI: body mass index; GFR: glomerular filtration rate; CRP: C-reactive protein; hsTnI: high-sensitive troponin I; CAD: coronary artery disease; CHF: congestive heart failure; ACE-I: angiotensin-converting enzyme inhibitor; ARB: angiotensin receptor blocker; ASA: acetylsalicylic acid; DOAC: direct oral anticoagulants.

**Table 2:** Composition of ECG score.

Score Parameter	% (*n*)
Contiguous Q waves	4 (5)
QRS duration	
≤80 ms	18 (24)
80–110 ms	69 (93)
>110 ms	13 (17)
LV hypertrophy	13 (18)
Prolonged JTc	31 (41)

**Table 3:** Sensitivity analysis for the prediction of cardiotoxicity.

Variable	Low Risk vs. High Risk	
	Value	95% CI
Sensitivity	0.82	0.74–0.88
Specificity	0.82	0.64–0.92
Positive predictive value	0.95	0.88–0.98
Negative predictive value	0.55	0.40–0.69

Abbreviations: CI: confidence interval.

**Table 4:** Risk group characteristics.

Parameters	Low Risk (*n* = 92)	High Risk (*n* = 42)	*p* Value
Age, years	61 ± 15	63 ± 14	0.4390
Female sex (%)	57	43	0.1418
BMI (kg/m^2^)	26.0 ± 4.4 **	29.1 ± 5.8	0.0050
LV-EF (%)	59 ± 6	57± 7	0.1722
Hemoglobin, g/dL	12.9 ± 1.9	12.5 ± 1.9	0.4897
Platelets, ×1000/µL	280 ± 110	269 ± 109	0.0664
Creatinine mg/dL	0.87 ± 0.23	1.06 ± 0.34	0.1578
CRP mg/dL	7.6 ± 2.0 *	2.2 ± 4.7	0.0308
hsTnI ng/L, (IQR)	0 (0–5)	0 (0–9)	0.0929
NT-proBNP pg/mL, (IQR)	128 (60–198)	160 (64–818)	0.2629
NYHA class (I/II/III/IV) (%)	74 **/25/1 **/0	45/41/14/0	
Comorbidities			
Arterial hypertension (%)	51	61	0.2434
Diabetes (%)	14	17	0.7023
Atrial fibrillation (%)	6	9	0.5396
Known CAD (%)	12	17	0.5770
Medication			
ACE-I/ARB (%)	41	52	0.2316
Betablocker (%)	29	57	0.0021
ASA (%)	26	43	0.0951
DOAC (%)	9	9	0.2408

All values are the mean ± SD unless indicated otherwise. Abbreviations: BMI: body mass index; LV-EF: left ventricular ejection fraction; hsTnI: high-sensitive troponin I; GFR: glomerular filtration rate; CRP: C-reactive protein; CAD: coronary artery disease; CHF: congestive heart failure; ACE-I: angiotensin-converting enzyme inhibitor; ARB: angiotensin receptor blocker; ASA: acetylsalicylic acid; DOAC: direct oral anticoagulants. ** *p* < 0.01 vs. high risk. * *p* < 0.05.

**Table 5:** Spearman rank correlation analysis.

Parameters	ECG Score		
	r	95% CI	*p*
Age (years)	0.0147	−0.1602–0.1888	1.0
Sex (F)	−0.2358	−0.3941–−0.0639	0.0915
BMI (kg/m^2^)	0.2274	0.0244–0.4124	0.3645
Heart rate (bpm)	−0.0216	−0.1953–0.1535	1.0
Presence of CAD	0.0999	−0.0766–0.2703	1.0
Presence of hypertension	0.1135	−0.0629–0.2830	1.0
Presence of diabetes	0.0501	−0.1262–0.2233	1.0
Presence of dyslipidemia	0.1653	−0.0101–0.3309	0.8580
Presence of atrial fibrillation	0.0301	−0.1465–0.2048	1.0
Betablocker intake	0.2290	0.0554–0.3891	0.1245
ACE-I/ARB intake	0.0723	−0.1049–0.2450	1.0
NYHA class (I–IV) *	0.2579	0.0853–0.3416	0.0435
hsTnI (ng/L)	0.0961	−0.0923–0.2779	1.0
NT-proBNP (pg/mL)	0.1530	−0.0427–0.3374	1.0
LV-EF (%)	−0.0911	−0.2667–0.0904	1.0

Abbreviations: BMI: body mass index; CAD: coronary artery disease; ACE-I: angiotensin-converting enzyme inhibitor; NYHA: New York Heart Association; hsTnI: high-sensitive Troponin I; LV-EF: left ventricular ejection fraction. * Scores increase per the NYHA class increase.

## Data Availability

The data presented in this study are available upon request from the corresponding author. The data are not publicly available due to ethical and legal issues.

## Supplementary Materials

The following are available online at https://www.mdpi.com/2072-6694/13/6/1197/s1, Table S1: In- and exclusion criteria, Table S2: Applied anti-tumor therapies.
