# Supplementary material for: ECG Scoring for the Evaluation of Therapy-Naïve Cancer Patients to Predict Cardiotoxicity
Source: Cancers (Basel). 2021 Mar 10;13(6):1197. doi: 10.3390/cancers13061197 (PMC7999575; doi:10.3390/cancers13061197)
Supplement: Supplementary file 1 [file cancers-13-01197-s001.pdf]

Supplementary Material: ECG Scoring for the Evaluation of Therapy-Naïve Cancer Patients to Predict Cardiotoxicity

Julia Pohl, Raluca-Ileana Mincu, Simone M. Mrotzek, Reza Wakili, Amir A. Mahabadi, Sophia K. Potthoff, Jens T. Siveke, Ulrich Keller, Ulf Landmesser, Tienush Rassaf, Markus S. Anker, Matthias Totzeck

Table S1. In- and exclusion criteria.

Inclusion Criteria	Exclusion Criteria
- Presentation in cardio-oncology before start of anti-cancer therapy<br>- Presentation in cardio-oncology for a follow-up routine	- Prior cancerous condition with anti-cancer therapy

Table S2. Applied anti-tumor therapies.

Applied Anti-Tumor Therapy	% of the Patients
Anthracyclines (%)	27%
Doxorubicin (mean dose (mg/m^2))	209
Epirubicin (mean dose (mg/m^2))	318
Combination therapy with alkylating agents	92%
Antimicrotubule agents (%)	14%
Platin-containing therapies (%)	4%
Antimetabolites (%)	1%
Topoisomerase II inhibitors (%)	13%
Tyrosine kinase inhibitors (%)	3%
Immune checkpoint inhibitors (%)	41%
BRAF/MEK inhibitors (%)	7%
Anti-Her2 compounds (%)	4%
Others (%)	16%
